# Alveolar hypoxia, alveolar macrophages, and systemic inflammation

**DOI:** 10.1186/1465-9921-10-54

**Published:** 2009-06-22

**Authors:** Jie Chao, John G Wood, Norberto C Gonzalez

**Affiliations:** 1 Department of Molecular and Integrative Physiology, University of Kansas Medical Center, Kansas City, KS 66160, USA

## Abstract

Diseases featuring abnormally low alveolar PO_2 _are frequently accompanied by systemic effects. The common presence of an underlying inflammatory component suggests that inflammation may contribute to the pathogenesis of the systemic effects of alveolar hypoxia. While the role of alveolar macrophages in the immune and defense functions of the lung has been long known, recent evidence indicates that activation of alveolar macrophages causes inflammatory disturbances in the systemic microcirculation. The purpose of this review is to describe observations in experimental animals showing that alveolar macrophages initiate a systemic inflammatory response to alveolar hypoxia. Evidence obtained in intact animals and in primary cell cultures indicate that alveolar macrophages activated by hypoxia release a mediator(s) into the circulation. This mediator activates perivascular mast cells and initiates a widespread systemic inflammation. The inflammatory cascade includes activation of the local renin-angiotensin system and results in increased leukocyte-endothelial interactions in post-capillary venules, increased microvascular levels of reactive O_2 _species; and extravasation of albumin. Given the known extrapulmonary responses elicited by activation of alveolar macrophages, this novel phenomenon could contribute to some of the systemic effects of conditions featuring low alveolar PO_2_.

## Introduction

Reduced alveolar PO_2 _is observed in a number of clinical settings, and is frequently associated with systemic effects, many of which present an inflammatory component. On the other hand, alveolar macrophage-induced systemic inflammation has been documented in humans and in animal experiments. The objective of this review is to describe a novel phenomenon, namely the systemic inflammation initiated by alveolar macrophages activated by a reduction of alveolar PO_2_. Investigation of the links between alveolar macrophages, alveolar hypoxia, and systemic inflammation could provide insights into the pathogenesis of the systemic effects of conditions associated with alveolar hypoxia.

### Systemic effects in conditions exhibiting low alveolar PO_2_

Systemic effects are frequently observed in pulmonary or extrapulmonary diseases associated with low alveolar PO_2_. While the pathogenesis of this diverse group of conditions is varied, the presence of systemic markers of inflammation has been demonstrated either in clinical cases or in animal models. Examples of systemic consequences of alveolar hypoxia in which an inflammatory component has been proposed are the cachexia and muscle wasting of chronic obstructive pulmonary disease [1-4], the insufficient hemopoietic response in pulmonary fibrosis [5], the cardiovascular and metabolic dysfunctions in sleep apnea [6-9], the multiple organ failure secondary to atelectasis [10], acute lung injury [11-13] and pulmonary contusion [14], the systemic inflammation of pneumonia [15,16] and the acute illnesses of high altitude [17-19]. While it is possible that systemic inflammation does not play a causal role in every one of these conditions, it is reasonable to assume that, at least, inflammation influences their development and outcome. Accordingly, a better understanding of the pathophysiological role of systemic inflammation should help in the management of conditions associated with alveolar hypoxia.

### Systemic effects of alveolar macrophage activation

There is evidence that alveolar hypoxia induces lung inflammation, and that alveolar macrophages play an important role in the modulation of this phenomenon. Rats breathing 10% O_2 _for periods ranging from 1 to 8 h show extravasation of albumin and increased pulmonary expression of HIF-1α, NF-κB, and pro-inflammatory cytokines; these markers are attenuated by elimination of alveolar macrophages [20-23]. Hypoxia leads to upregulation of the expression of neurokinin-1 receptors in alveolar macrophages and in epithelial cells [24]. Activation of these receptors leads to inflammatory responses mediated by cytokines IL-1, IL-6, and TNFα [24,25]. Furthermore, alveolar macrophages have been implicated in the synergistic effects of hypoxia on pathogen-induced lung inflammation [26,27].

In addition to the well known pulmonary effects of alveolar macrophage activation with hypoxia and other stimuli, there is mounting evidence that activation of alveolar macrophages may have substantial extrapulmonary effects. An example is the systemic microvascular response to particulate matter inhalation. Epidemiological studies have demonstrated a correlation between environmental air pollution and cardiovascular morbidity [28], and human and animal studies have shown that phagocytosis of fine particles by alveolar macrophages leads to pulmonary inflammation with increased number of activated alveolar macrophages [29]. This is accompanied by elevated levels of circulating cytokines, systemic inflammation, and microvascular endothelial dysfunction in the systemic circulation [30-33]. It has been suggested that following phagocytosis of particulate matter, cytokines released by activated alveolar macrophages act on the bone marrow to mobilize platelets and leukocytes which stimulate the release of acute phase proteins and lead to systemic inflammation [34].

The results discussed below will show that reduction of alveolar PO_2 _activates alveolar macrophages and initiates a systemic inflammatory cascade, demonstrating the presence of a link between alveolar hypoxia, alveolar macrophages and systemic inflammation.

### Alveolar hypoxia and systemic inflammation

Rats breathing 10% O_2 _show a rapid inflammatory response in mesentery, skeletal muscle and pial microcirculations within minutes of the onset of hypoxia [35-38]. This response is characterized by increased levels of reactive O_2 _species (ROS) [39], mast cell degranulation [40], increased leukocyte-endothelial adhesive interactions [35-37,40], and extravasation of albumin [41]. Increased levels of ROS-dependent fluorescence occur within minutes of the onset of hypoxia, and are observed in perivascular mast cells, in the endothelial layer of postcapillary venules and at the sites of leukocyte-endothelial adherence [39,41]. The magnitudes of the ROS-dependent fluorescence intensity, and of the leukocyte-endothelial adhesive interactions are inversely related to the PO_2 _value [42]. Both ROS-dependent fluorescence intensity and increased leukocyte-endothelial adherence were significantly attenuated by the antioxidants SOD/catalase and lipoic acid [39,41]. Increasing microvascular NO levels by administration of a NO donor, spermine NOnoate (sNO) or of the NO precursor L-arginine blocked the increases in ROS and in leukocyte-endothelial interactions induced by hypoxia [42]. This suggests that hypoxia is associated with a decrease in NO as well as an increase in ROS levels. NO levels could be reduced as a result of consumption by the elevated ROS; alternatively, NO generation could be decreased by reduced NO synthase (NOs) activity due to limitation of O_2 _substrate availability in hypoxia [43,44]. However, if this were the case, it would be expected that administration of the substrate L-arginine would not be effective in restoring NO levels during hypoxia. The observation that administration of L-arginine and of sNO had the same effects, qualitatively and quantitatively, suggests that the decrease in microvascular NO levels is not the result of reduced NO synthesis, but of increased consumption by ROS[42].

Mast cell degranulation is a necessary event which provides the chemotactic gradient for the increased leukocyte-endothelial adhesive interactions of hypoxia [40]. Prevention of mast cell degranulation with cromolyn, a mast cell stabilizer, attenuates all of the markers of inflammation [36,40]. The inflammatory cascade includes activation of the local renin-angiotensin system (RAS): the leukocyte-endothelial adherence and increased vascular permeability observed in skeletal muscle during alveolar hypoxia are attenuated by inhibition of angiotensin converting enzyme (ACE) and by blockade of angiotensin II (Ang II) receptors [45].

A series of observations suggests that the key initial event of the inflammatory response, the activation of mast cells, is not triggered by the reduced PO_2 _of the environment surrounding the mast cells, but rather by an agent(s) released by alveolar macrophages into the circulation. The evidence leading to this hypothesis is as follows:

#### Selective reduction of tissue microvascular PO_2 _does not induce inflammation unless it is accompanied by alveolar hypoxia

Cremaster microvascular PO_2 _(C*mv*PO_2_), estimated using a phosphorescence decay method [36], was selectively reduced in rats breathing room air. Cremaster hypoxia was induced either by mechanical restriction of cremaster blood flow [36], or by *in vivo *equilibration of the cremaster with 5% CO_2_/95% N_2 _[36,37] in the presence of normal systemic arterial and alveolar PO_2_. Although C*mv*PO_2 _decreased to levels comparable to those seen in rats breathing 10% O_2_, neither of these interventions produced mast cell degranulation or leukocyte endothelial adherence in cremaster post-capillary venules. On the other hand, cremaster mast cell degranulation and leukocyte adherence occurred when the animals breathed 10% O_2 _and C*mv*PO_2 _was maintained at a higher than normal level [36,37]. One possible explanation for these results, among other alternatives, is that mast cell degranulation is triggered by an agent released from a distant site.

#### Plasma from hypoxic rats induces inflammation in normoxic tissue

If a putative mediator released from a distant site is transported by the systemic circulation, it would be expected that plasma obtained from hypoxic animals would elicit inflammation in normoxic tissues. Plasma obtained from conscious rats breathing 10% O_2 _for 5 min produced mast cell degranulation, leukocyte endothelial adherence, and extravasation of albumin when applied to the cremaster muscle of normoxic rats [46]. The inflammatory effect is specific for hypoxic rat plasma since plasma from normoxic animals had no effect. The inflammation is not triggered by mediators released from activated mast cells or adherent leukocytes into the plasma of the donor rat: pretreatment of the donor with cromolyn, which blocks alveolar hypoxia-induced mast cell degranulation and leukocyte adherence [40], did not attenuate the response to hypoxic rat plasma. The inflammatory agent contained in hypoxic rat plasma is not originated in blood cells, since plasma separated from blood equilibrated *in vitro *with hypoxic gas mixtures did not produce inflammation [46].

#### Alveolar macrophages are necessary for the inflammation of alveolar hypoxia

Since systemic inflammation occurred only when alveolar PO_2 _was reduced, alveolar macrophages, given their location and their systemic effects, were thought of as a likely source of the circulating mediator of the systemic inflammation of alveolar hypoxia. A role for alveolar macrophages in this phenomenon was demonstrated by three lines of evidence [47]: first, depletion of alveolar macrophages by tracheal instillation of clodronate-containing liposomes blocked the mast cell degranulation, the increased leukocyte-endothelial adherence and the extravasation of albumin that follows alveolar hypoxia in intact rats. Second, plasma obtained from hypoxic, alveolar macrophage-depleted rats did not elicit inflammation when applied on the normoxic cremaster; third, supernatant of primary cultures of alveolar macrophages exposed to 10% O_2 _induced mast cell degranulation and leukocyte endothelial adherence when applied topically onto the normoxic cremaster. A non-specific effect was ruled out by the observation that supernatant of alveolar macrophages cultured in normoxia had no inflammatory effect. The inflammation initiated by hypoxic alveolar macrophage supernatant shares common pathways with that secondary to alveolar hypoxia in intact animals, since both are attenuated by blockade of the RAS [45,47].

#### Mast cell degranulation leads to activation of the local RAS

Non-selective Ang II antagonists and ACE inhibitors attenuate the inflammation induced by alveolar hypoxia in intact rats, pointing to a participation of the RAS in the inflammatory cascade initiated by alveolar hypoxia. Several lines of evidence obtained in skeletal muscle and mesentery microcirculations indicate that mast cell degranulation is responsible for the activation of the RAS: first, while cromolyn blocks the inflammatory effects of hypoxic rat plasma and of hypoxic alveolar macrophage supernatant, it does not block the inflammatory response to topical Ang II [45]. This is in agreement with the observation that topical Ang II produces increased leukocyte-endothelial adherence and increased albumin extravasation, but does not cause mast cell degranulation [45,48]. Finally, the leukocyte-endothelial adherence induced by the mast cell secretagogue C4880 is attenuated by ACE inhibition and by Ang II receptor blockade [45,48]. While it is clear that the RAS is activated by mast cells, the underlying mechanism is uncertain. Mast cells of some species contain chymases which act as ACE to convert Ang I to Ang II [49]; alternatively, renin contained in mast cells [50,51] may initiate the RAS cascade. Activation of the RAS by cardiac mast cell renin released during ischemia/reperfusion has been demonstrated recently [51]. It is important to keep in mind that while the role of the RAS in the inflammation of alveolar hypoxia is strongly supported by the data presented here, these results do not rule out the participation of additional mast cell-borne inflammatory mediators, such as histamine.

Supernatant of hypoxic alveolar macrophages elicits degranulation of mast cells followed by activation of the RAS in two different microvascular beds: mesentery and skeletal muscle. The similarity of these responses is consistent with the notion of a widespread inflammation originated by an alveolar macrophage-borne mediator carried by the circulatory system.

**Figure 1:**
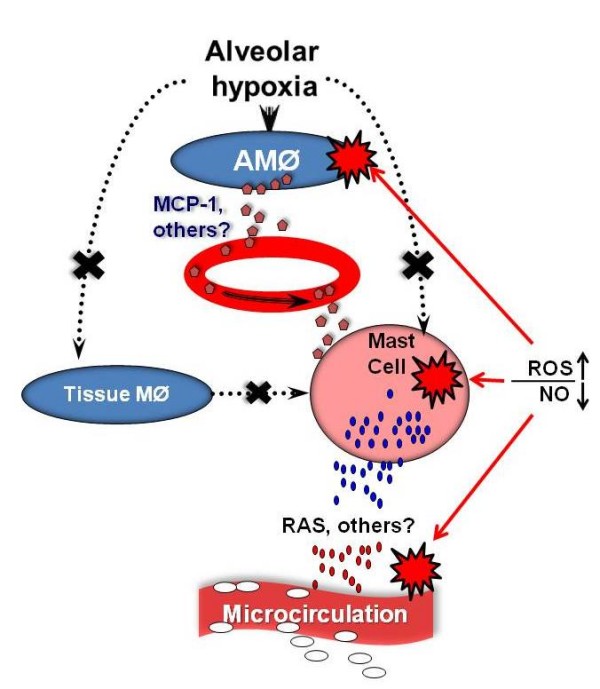
**Schematic representation of the systemic inflammation of hypoxia**. Reduced alveolar PO_2 _activates alveolar macrophages (AMØ) but not peripheral tissue resident macrophages (Tissue MØ) or mast cells. Activated AMØ release H_2_O_2 _which is the product of dismutation of O_2_^- ^generated during the respiratory burst. AMØ stimulation leads to release of monocyte chemoattranctant protein-1 (MCP-1), a chemokine with mast cell secretagogue properties. Hypoxia-induced release of additional mediators by AMØ can not be ruled out. The mediator is trasported by circulation and activates mast cells. Activation of mast cells is evidenced by degranulation and by generation of reactive O_2 _species (ROS) and reduction of NO levels. Mast cell activation leads to microvascular inflammation charcterized by increased leukocyte-endothelial adhesive interactions, leukocyte emigration and increased vascular permeability. The renin-angiotensin system (RAS) is activated by mast cell degranulation and participates in the production of the inflammation, although participation of other mast cell-borne mediators (histamine) can not be ruled out. ROS generation is detected in the endothelial layer of post-capillary venules as well in the sites of adhesion of leukocytes.

The results presented so far suggest that reduction of alveolar PO_2 _in intact organisms initiates the sequence of events presented schematically in Figure 1: alveolar macrophages activated by hypoxia release a mediator into the circulation; this mediator activates mast cells which, in part at least through activation of the RAS, induce systemic inflammation. If this sequence of events is correct, several conditions must apply:

##### Alveolar macrophages, but not resident tissue macrophages, are directly activated by low PO_2_

Indirect evidence that alveolar macrophages are activated by hypoxia is provided by the observation that supernatant of hypoxic alveolar macrophages initiates an inflammatory response in skeletal muscle [47] and in mesentery [48]. More direct evidence of hypoxia-induced activation was provided by studies in primary cultures of alveolar and peritoneal macrophages, and peritoneal mast cells. In these studies, the cell cultures were equilibrated with gas mixtures providing a range of PO_2 _values that would encompass conditions from normoxia to severe hypoxia *in vivo*. In this respect it should be remembered that alveolar macrophages are normally exposed to PO_2 _values close to 100 Torr, while resident tissue macrophages (and tissue mast cells) may be in "normoxic" conditions in PO_2 _environments lower than 30–35 Torr [48]. Equilibration of alveolar macrophages with hypoxic gas mixtures (PO_2 _ranging from 5 to 65 Torr) resulted in a respiratory burst evidenced by a release of H_2_O_2 _into the supernatant, the magnitude of which was inversely related to the medium PO_2_. The H_2_O_2 _release reached a peak at 15 min of hypoxia and returned to pre-hypoxic values by 30 min of exposure [48]. Equilibration of alveolar macrophages with PO_2 _> 100 Torr did not induce a respiratory burst.

In contrast with alveolar macrophages under hypoxia, peritoneal macrophages did not release H_2_O_2 _when medium PO_2 _was reduced to values as low as ~5 Torr. The dissimilar effects of hypoxia on activation of the two types of macrophages are paralleled by the different effects of their supernatants applied onto the normoxic mesentery: while supernatant of alveolar macrophages exposed to hypoxia for > 30 min elicited mast cell degranulation and leukocyte endothelial adherence, supernatant of peritoneal macrophages equilibrated with even lower PO_2 _had no inflammatory effects [48]

##### Mast cells are not directly activated by low PO_2_, but they degranulate when in contact with hypoxic alveolar, but not with resident tissue macrophages

According to the sequence of events illustrated in Figure 1, mast cells are activated by a mediator released by hypoxic alveolar macrophages, and not by the reduced local tissue PO_2_. That this is the case was suggested by the lack of cremaster mast cell degranulation in the experiments in which C*mv*PO_2 _was selectively reduced while alveolar and arterial PO_2 _were maintained within normoxic values [36,37]. These results were confirmed by in vitro experiments: reduction of medium PO_2 _to ~5 Torr failed to elicit degranulation in primary cultures of peritoneal mast cells. However, degranulation occurred when mast cells of the same culture were immersed in supernatant of alveolar macrophages which had been exposed to a PO_2 _of ~65 Torr for 30 min. In contrast, immersion of peritoneal mast cells in supernatant of hypoxic peritoneal macrophages (PO_2 _~5 Torr) did not elicit degranulation. These results provide a direct link between alveolar macrophages and mast cells and demonstrate that alveolar macrophages release a mast cell secretagogue when stimulated by hypoxia. The results also show that reduced tissue PO_2 _and activation of resident tissue macrophages are not necessary to initiate the systemic inflammation of alveolar hypoxia.

##### Alveolar macrophages, but not peritoneal macrophages or mast cells release a mast cell secretagogue during hypoxia

The hypothesis that the systemic inflammation of alveolar hypoxia is initiated by an alveolar macrophage-borne mediator is strengthened by the findings that monocyte chemoattractant protein-1 (MCP-1), a mast cell secretagogue, is released by primary cell cultures of alveolar macrophages exposed to hypoxia [48]. MCP-1, a chemokine of the CC family, was the only agent of several investigated which demonstrated a several-fold increase in the supernatant of alveolar macrophage cultures 30 min after reduction of the PO_2_. In contrast, no changes in MCP-1 supernatant concentration were observed when primary cultures of peritoneal macrophages or peritoneal mast cells were exposed to hypoxia [48]. MCP-1 fits the criteria for a putative mediator of hypoxia-induced inflammation: MCP-1 induces chemotaxis of alveolar macrophages, mast cells, and human T-lymphocytes [52]. MCP-1 is released from alveolar macrophages *in vitro *in response to hypoxia and hypoxia/reoxygenation [53,54], influences distal organ damage in hemorrhagic shock [55] and activates mast cells to elicit microvascular inflammation [56-58]. Further studies are necessary to determine the mechanism underlying the release of MCP-1 by alveolar macrophages, the interaction of MCP-1 with mast cells, and whether other alveolar macrophage-borne agents participate in the activation of mast cells. Mast cell secretagogues potentially released from alveolar macrophages include neuropeptides such as adrenomedullin, CGRP, and substance P [24,25,59]. These proinflammatory agents have several physiological functions [58] and participate in inflammatory processes, including pulmonary responses to hypoxia and sepsis [27,59].

### The lung as a target of systemic hypoxia or ischemia

This review addresses a novel phenomenon, the systemic inflammation elicited in response to activation of alveolar macrophages, the stimulus in this case being a reduction of alveolar PO_2_. The opposite phenomenon, namely acute lung injury initiated by remote events, has been known for some time and is exemplified by the acute lung injury which may develop after non-thoracic trauma, hemorrhage, sepsis, or ischemia/reperfusion[60]. A frequent example is the acute lung injury secondary to intestinal ischemia/reperfusion (I/R) that follows hemorrhagic shock and resuscitation. The pulmonary response in this case is characterized by leukocyte recruitment, alveolar macrophage activation, endothelial and epithelial cell damage, increased vascular permeability and pulmonary edema [61]. Both the systemic effects of alveolar macrophage activation and lung injury secondary to intestinal ischemia/reperfusion-feature remote inflammatory responses elicited by a mediator transported from a distant site. However, while the results presented here indicate that the mediator released by hypoxic alveolar macrophages is transported by blood, mounting evidence suggests that the agent(s) responsible for the lung injury secondary to intestinal I/R is transported by mesenteric lymph. Diversion of mesenteric lymphatic outflow blocked the acute lung injury following shock/resuscitation [62], and mesenteric lymph, but not portal venous blood of rats undergoing hemorrhagic shock increased permeability of isolated endothelial cell monolayers and induced acute lung injury [63]. Mesenteric lymph from shocked rats primed neutrophils for production of superoxide, increased expression of surface adhesion molecules, and inhibited leukocyte apoptosis [64]. Further research demonstrated that the agent responsible for this effect is contained in the lipid fraction of lymph [65]. Recent observations suggest an involvement of arachidonic acid as a mediator of the lung injury following intestinal ischemia/reperfusion. According to this scenario, ischemia/reperfusion would activate phospholipase A2 to release arachidonic acid into the lymph. Arachidonic acid, in turn, would contribute to the initiation of the inflammation and the local generation of leukotriene B4 in the lung [65,66].

Although there is evidence that alveolar macrophage activation plays an important role in the development of acute lung injury induced by intestinal I/R, its exact role is not clear. Alveolar macrophages recovered from broncho alveolar lavage of rats with intestinal ischemia reperfusion release more H_2_O_2 _in response to phorbol myristate acetate and produce more TNFα than those recovered from control animals[67]. Selective alveolar macrophage depletion with intratracheal instillation of clodronate-containing liposomes significantly attenuates the increase in pulmonary vascular permeability of rats with intestinal ischemia/reperfusion [68]. Hemorrhagic shock is associated with an increase in LPS-induced TNFα and a decrease in the anti-inflammatory cytokine IL-10 by alveolar macrophages [69], as well as LPS-induced nuclear translocation of NF-KB in alveolar macrophages. All these markers of activation are attenuated in alveolar macrophages recovered in broncho alveolar lavage fluid of hemorrhagic shock rats resuscitated with hypertonic saline, a treatment which prevents hemorrhagic shock-induced lung injury [70].

Thus, while the lungs can be the target of remote ischemic or hypoxic processes, they also can be the source of agents that may induce inflammatory responses in peripheral tissues. While the specific cellular pathways and the modes of translocation of inflammatory agents vary in different conditions, these examples point out to an important phenomenon, namely the production of inflammatory responses initiated from remote sites, an issue of important clinical significance.

## Conclusion

In summary, strong evidence supports the hypothesis that the systemic inflammation of alveolar hypoxia is initiated by the release of a circulating mediator from activated alveolar macrophages. The data represents an example of a growing body of evidence regarding systemic effects of alveolar macrophage activation. While the sequence of events described in this review has firm experimental support, several areas remain unclear. These include the mechanism of activation of the alveolar macrophages, the possible contribution of additional alveolar macrophage-borne mediators, the mechanism of activation of the RAS, and whether Ang II is the only effector of the microvascular response. It should be kept in mind that exposure of an intact organism to environmental hypoxia is a complex stimulus which sets in motion a number of processes with different time courses. Other mechanisms are likely to participate later in the development of the systemic inflammation. Nevertheless, given the widespread nature of the inflammatory response, and the prevalence of inflammation in many conditions associated with alveolar hypoxia, further understanding of this phenomenon should provide insights into the role of inflammation in conditions associated with reduced alveolar PO_2_.

## Competing interests

The authors declare that they have no competing interests.

## Authors' contributions

JC performed the experiments in intravital microscopy and primary cell lines described in reference 48 and edited the manuscript; JGW edited the manuscript and NCG wrote the manuscript. All authors read and approved the final version of the manuscript.
